# Digital (mis)trust: ethnographic encounters with computational forms

**DOI:** 10.1080/17530350.2024.2407853

**Published:** 2024-11-12

**Authors:** James Maguire, Kristoffer Albris

**Affiliations:** a IT University of Copenhagen, Technologies in Practice Research Group, Copenhagen, Denmark; b University of Copenhagen, Copenhagen Centre for Social Data Science and Department of Anthropology, Copenhagen, Denmark

**Keywords:** Trust, mistrust, digital, computational, machinic, mutations

## Abstract

This special issue is driven by a curiosity about the role of computational forms – practices, logics, technologies, and infrastructures – in social life and how they mediate issues of trust and mistrust: designated *(mis)trust*. Through a series of ethnographic encounters, its contributors describe how (mis)trust is rendered as an issue of concern by various actors as it is problematized, conceptualized, narrated, and designed for. In doing so, the papers analyse the work (mis)trust does in these settings, with a focus on the role of computational thinking within public discourses on democratic elections, the use of computational technologies in establishing bureaucratic order, the computational practices at play in the production of coding subjectivities, and the computational artefacts that assure data circulations within digital infrastructures. This introduction argues for a more expansive understanding of the relation between trust and mistrust in the digital age, countering the oftentimes default rendering of these concepts as antonymic. Instead, it argues that they live in a mutable relation. Despite prevailing technosolutionist approaches to (mis)trust, it cannot, we suggest, be solved for, resolved, or even eviscerated. Whatever actors (engineers, programmers, professionals etc) do in their efforts to ‘fix’ mistrust, it continues to mutate as a social form.

## 1. Introduction

As digital technologies, platforms, and infrastructures creak and moan under the weight of serial misuse, the clarion call to regain trust in technology has never been more resounding. And while citizens and institutions continue to figure out how to live with these technologies after more than two decades of digital transformation optimism, this special issue turns its analytical attention to some of the ways that trust and mistrust – designated *(mis)trust* – figure in these concerns. In particular, we are drawn to the question of how (mis)trust animates social life under emerging conditions of computation digitalization. In other words, we open an analytical space that reflects upon the role of computation computational forms (practices, logics, technologies, and infrastructures) in mediating relations of (mis)trust amongst various types of actors (citizens, experts, students, and the state).

The contributors to the special issue describe, amongst other things, various instances of unease around if, who, what, and how we can trust in an increasingly digitalized world. Contemporary examples are rife, be it perceived disinformation threats to democratic processes, the health and welfare of children and young adults exposed to digital technologies, the surveillance concerns of citizens and organizations from digital platforms, cyber security efforts to design safe encryption, or the panoply of issues emerging under the banner of generative AI. (Mis)trust, it strikes us, has become the rhetorical ground upon which many forms of such digital unease play out.

This special issue gathers a collective of anthropologists and Science and Technology Studies (STS) scholars conducting research at the interface of society and the digital, particularly around an array of socio-political issues emerging from datafication, coding, cybersecurity, and democracy. What their work makes clear is that questions of (mis)trust frequently reside within the orbit of other, more explicit, concerns about digital process; be it the issue of cybersecurity *assurance* (Spencer) the *promises* of digital democracy (Gad), the production of coding *subjectivities* (Breslin), or the *formation* of a citizenry under intensive processes of datafication (Singh). Yet the forms that (mis)trust takes in such cases are highly variable and context specific. As such, this special issue offers a set of insights into such forms, the conditions under which they arise (their histories and practices), the conflicts they embed and engender (their politics), and the relationships that maintain or constrain them (their infrastructures).

## 2. Problematizing trust and mistrust

While there is little agreement within academia, and beyond, as to the precise meaning of the term *trust* (Bauer 2015), there is broad consensus as to its importance across various societal scales; be relations between states, within communities, institutions, and organizations, or the plethora of interpersonal relationships that are formative of everyday practices. In policy circles, trust is said to enhance our ability to make agreements with one another, to uphold contractual arrangements, to promote self-expression, to reduce crime, and even to generate more happiness (Andreasson 2017). In welfare states, in particular, trust is seen as a key societal resource to be nurtured and protected in the maintenance of welfare standards (Kumlin, Stadelmann-Steffen, and Haugsgjerd 2018; Schou and Hjelholt 2017). In political discourse, trust has become an object of rigorous measurement and an important rhetorical asset as national and international indexes become quasi surrogates for more clearly delineated political action (Bauer and Freitag 2018). As a result, measuring the general level of trust between and within groups and institutions in society is increasingly seen as a primary health indicator of the body politic (Brownlie, Greene, and Howson 2008). In many of these accounts, trust is discursively presented as an omnipresent social form. One that, while oftentimes problematized and politicized, is difficult to conceptualize or analyse. Nonetheless, the social sciences have invested much effort in attempting to do just that.

In economics, trust is broadly seen as a way to reduce complexity and facilitate smoother exchanges of information in markets (Gambetta 1988; Williamson 1993). Political scientists talk of trust in various modes; institutional, social, and political (Newton and Zmerli 2011). In particular, they focus on the role of trust in legitimizing elections, institutions, and state-building projects, as well as on how such trust varies across different socio-political groups (Rose et al. 2013). In sociology, trust is integral to ongoing discussions about social cohesion and social capital (Hooghe 2007; Putnam 2000; Simmel 1950) and the distribution of power between and within groups in society (Luhmann 2018; Seligman 2000). In particular, there is a strong line of sociological thinking that associates trust with a variety of key social outcomes, such as reciprocity, collective action, solidarity, equality, and social order (Schilke, Reimann, and Cook 2021, 240).

Much work in anthropology and STS (our home disciplines) regularly incorporates more critical and feminist perspectives (Baier 1986; Ballestero 2023; Broch-Due and Ystanes 2016; Bruun, Andersen, and Mannov 2020; Carey 2017; Mühlfried 2018; Weichselbraun, Galvin, and McKay 2023; Yanagisako 2002). A key aim of much of this literature is to challenge some of the relatively *thin* articulations of social relations – their various power implications, hierarchies, complexities, and intricacies – that have emerged from trust discourses within more dominant strands of social science. Anthropology regularly offers sets of ethnographically sensitized accounts that trace the multitude of relationships, conflicts, structures, and histories that constitute (mis)trust in any given setting (Biruk 2023; Zhang 2023). While space does not permit us to develop much of this, one of the more pronounced critiques attempts to combat an over-commitment to epistemological individualism (Broch-Due and Ystanes 2016) and its concomitant transactional and contractual articulations of trust. In such renderings, trust is oftentimes seen as a cognitive assessment of the trustworthiness of others: a form of knowledge upon which ‘trustors’ and ‘trustees’ evaluate if their interests are encapsulated by the interests of those they are transacting with (Hardin 2004; 2006). In these accounts, the sphere of the intimate (home, family, and kin) becomes the primary terrain upon which trusting relations can be built and maintained. In contrast to this, building trusting relations with putative ‘strangers’ is the provenance of institutional arrangements; the adoption of overlapping sets of complex, and mutually interdependent, institutional networks of checks and balances, purportedly produce trust through internal governance mechanisms, external control, and the division of power (Cook, Hardin, and Levi 2005; Giddens 1990; Shapiro 1987; Sztompka 1999). Critical anthropologies of trust (rightly) take issue which such renderings, insisting upon a more nuanced reading of the translations and overlaps between putatively public and private spheres. Intimate relationships, they suggest, are not merely refuges from the cold, rational calculus of the self-interested market but are also structured through power asymmetries and hierarchies (Broch-Due and Ystanes 2016).

As part of a more broadscale materialist turn within anthropology, recent work has shifted its analytical gaze towards the technologies and infrastructures through which trust is made, managed, or transformed (Bruun, Andersen, and Mannov 2020; Weichselbraun, Galvin, and McKay 2023). What this work demonstrates is a sensitivity to the manifold ways that trust emerges in oftentimes unstable and contradictory forms across various scales. Particular attention is given to how technologies and infrastructures of trust ‘assemble material objects, practices, logics, and modes of organization that enable broader projects of politics, market making, development practice, and social relations’ (Weichselbraun, Galvin, and McKay 2023, 4). Building upon scholars that have analysed trust as embedded within audit regimes and institutional economies of information, accountability and transparency (Jiménez 2011; Strathern 2000), such work homes in on the paradoxes that can arise in neo-colonial and extractive contexts where, for example, technologies of accountability oftentimes clash with more informal mechanisms of trust building (Weichselbraun, Galvin, and McKay 2023, 7–8).

So, while much work within social science still holds onto a particularly normative and modernist reading of trust as a meta category that underpins social life – or in fact, prescribes trust as the *sine qua non* of social order and cohesion – these latter critiques take issue with the dominant patriarchal logics of disciplines such as philosophy and economics, bringing questions of power, difference, and coloniality back into the equation. In particular, they push back at the idea of these disciples being the self-ascribed universalist windows onto how we think about trust and its centrality to the liberal imagination (Strathern 2021).

In conversation with the aforementioned research literatures, this special issue analyses four ethnographic encounters where various computational forms (practices, logics, technologies, and infrastructures) have become important mediation points for considering questions of (mis)trust in social life. In focusing on (mis)trust under emerging conditions of digitalization, our contributors turn their attention to these forms as mediation points within democratic institutions (Gad), subject formation (Breslin), bureaucratic order (Singh), and professional expertise (Spencer). And while we will probe issues of (mist)trust in digitally mediated relationships later in this introduction, in the following section we address our understanding of this somewhat cumbersome portmanteau, *(mis)trust*.

Working from within what we describe above as critical anthropologies of trust, anthropologists Florian Mühlfried (2018; 2019) and Matthew Carey (2017) warrant particular attention. While their critiques of the concept follow the contours of some of the ideas outlined above – the characterization of trust as an overarching normative, and at times virtuous, concept undergirding much of social life – they also turn critical attention to more common ways of conceiving mistrust. For Mühlfried, the default mode of rendering mistrust as oppositional to trust lends itself to a vision of the concept as a form of absence.

Associatively, such absences tend to be read through a moral prism as problematic. Here we see how mistrust is constituted as a *problem*: it constructs the mistrustful *other*. Mühlfried argues that a more productive interpretation might be to see mistrust as a strategy that is meaningful in particular contexts. One that works to make sense of difficult situations and engenders a sense of agency in actors beyond the purview of those in power (2018). Here Mühlfried reinterprets Niklas Luhmann’s oft cited reading of trust and mistrust as functionally equivalent ‘engagements with complexity' rather than merely ‘reductions of complexity.' This more capacious lens opens up an avenue for ethnographically investigating the different forms that (mis)trust can take. Here we see a rich ensemble of strategies and logics that mistrust is said to enrol and display, be it the drive to domesticate (for example, by offering strangers hospitality), or to distance (holding back resources or emotions in relationships).

Carey, on the other hand, offers a concept of mistrust that is less strategic and more dispositional; a way of thinking and acting in specific cultural constellations (2017, 7–8). While the western liberal imaginary of the person makes an assumption about the knowability of others – undergirding a set of shared assumptions about the nature of social relationships – this is not the case everywhere. Carey argues that in the Moroccan High Atlas the knowability of the other is not given and, hence, is not the pre-conditional ground upon which trusting relations can be developed. Carey, then, gives us a way of reading trust and mistrust along central epistemological fault lines, while binding them together conceptually as shadows: ‘for each implies its shadow: where people assume the other is known and so trusted, they are also aware that this sometimes does not hold; and where they assume that others are largely unknowable, they are also aware that some are less unknowable than others (2017, 10). Here we get an inclination as to *why* mistrust is so often characterized as an antonym of trust in the literature; its antonymic form becomes the necessary other to a particular set of westernized epistemic and onto-political concepts (knowability and alterity). And herein lies the richness of the term for us. Mistrust, as we read it, lives in a *mutable relationship* to trust: it can be constituted by (and constitute) trust, it can remain tacit in certain circumstances, it can surface occasionally, or episodically, or even endemically. In essence, it can emerge in various forms, through various strategies, with various effects, as it shadows with (Carey 2017; Jimenez 2011; Strathern 2011), and mutates in relation to, trust. We suggest the portmanteau (mis)trust to index such a mutable relationship.

## 3. (mis)trusting the digital

As Niklas Luhmann (1988) observed some time ago, societies tend to make trust an *issue* at particular historical junctures. When people are confronted by rapidly changing, and oftentimes inscrutable, landscapes, they tend to evoke trust as an idiom to say that important matters are at stake (Harper 2014, 311).^1^ But this evocation also matters; it can modify those landscapes, oftentimes opening the way for a consideration of ancillary practices and ideas that might otherwise go unnoticed (assurance, security, privacy, and so forth). Our proposition, then, is that the continued encroachment of computational forms (practices, logics, technologies, and infrastructures) into various scales of social life has triggered a plethora of concerns, many of which travel under the rhetorical banner of (mis)trust. Let us sketch out a few of these concerns here.

While trust in technology, and its relationship to knowledge and expertise, has been a long-standing area of social scientific inquiry – particularly within Science and Technology Studies (STS) (Jasanoff 2022; MacKenzie 1998) and the history of technology (Porter 1996) – the digital infrastructuring of everyday life has raised a series of renewed questions about trust as a contemporary social form. It is difficult to overstate the connection between the rise and power of Big-Tech corporations – in particular their datafication and colonization of digital spaces and practices (Couldry and Mejias 2020) – and the litany of digitally inflected controversies that emerge almost daily: disinformation, data breaches and cyber-attacks, the ethics and regulation of data, the role of predictive technologies, or, as we mentioned earlier, the surge in generative AI.^2^ And while the appearance of technology company executives performing mea culpas before democratically elected legislatures around the world – asking the public to, once again, trust them – has become old hat, a clear sense has emerged that the overly inflated democratic claims of internet 2.0 are difficult to maintain. Such claims are co-extensive with the very inception of the internet (the APARNET)^3^ where a particular figure of end users as benign, decent folks wanting to leverage computing for their own, mostly scientific, ends (Harper 2014, 300) has become the baseline trope that has fed the liberal imagination. We are not, it is safe to say, in Kansas anymore. And here we can identify one specific and important change that digital technologies have wrought. Echoing what Matthew Carey (in the afterword to this special issue) calls the rise of ‘identificatory mistrust’, the question is, it seems, less about the deceney or otherwise of the imagined end user, and more about how we never really *know* who the so-called end user actually is.

What arose from the ashes of the dot com bubble in the early 2000s – putting paid to the more open-protocol, anarchist-informed sensibility (Kelty 2008) of earlier internet imaginaries – was a digital landscape mediated by a host of institutional actors of varying scales. Central to the stabilization of this more econ-o-centric and transactional model of digitalization was the development of a ‘trusted third party payment system.’ As recounted by Nelms et al (2017), a group of transaction intermediaries (banks, regulatory, and other actors) became those ‘trusted’ to intervene in the economic and social lives of society’s actors (ibid), settling, as it were, the difficult issues around the ‘promise to pay’.^4^ What we see here is the emergence of particular *infrastructures of trust* (Bruun et al 2020; Nelms et al 2017; Weichselbraun et al 2023) engendered through a range of transactional standards and regulations, providing the basis of, and frame for, much of today’s digitalized social and economic actions. Later, more platformed, enactments of these intermediaries leveraged themselves as trust brokers, mediating the spaces between actors by attempting to produce trust as a design outcome. In a putative effort to make the history of transactions between market actors visible, so-called peer-to-peer platforms such as Airbnb, Uber, and eBay, subject actor exchanges to various forms of monitoring, datafication, and transparency (Bodo 2021, 2676). At stake here is a particular understanding of trust as a problem rendered solvable through design logics that correlate reputation with data, where reputation is (ideally) made visible through histories of data transactions. One that is materialized within digital infrastructures through selective protocols and tools. Here trust is reconfigured as an issue that can inhibit market relations. Mistrust, we might say, is the methodological innovation upon which such intermediation is premised.

However, in much the same way as one financial event (the dot com bubble) was implicit in the ascendence of these intermediaries, another (the 2008 financial crisis) has given rise to a wave of disintermediation technologies.^5^ The rise of Distributed Ledger Technologies (DLTs), in particular the blockchain, while attempting to reclaim some of the early internet’s more liberatory rhetoric, is at heart an engineering approach to questions of digital infrastructures, markets, and governance (Baldwin 2018; Dodd 2017; Howson 2020). Here, we see a move from trust as constituted through a relation between reputation and data to more compliance inflected forms. Putatively decentralized, distributed systems supplement trusted third parties with cryptographic systems that allow the verification and proof that certain trust assumptions are correct (Ruh 2018, 42). As such, non-compliance with sets of prescribed rules is prohibited through blockchain design protocols. So, while disintermediation technologies are materialized forms of mistrust (in banks, regulators, and the state) they are also forms of trust in designable, programmable, and hence, computational, approaches to sociality.

It is such developments around questions of trust in the digital age that puts it at the heart of ‘surveillance capitalism’ (Amoore 2020; Cheney-Lippold 2017; Doctorow 2020; Morozov 2013; Zuboff 2019). The effort to create datafied societies is shrouded in under-articulated risks where questions of monitoring and compliance slowly begin to supersede those of governance. For Amoore (2020) and Zuboff (2019) in particular, this represents a form of ‘machinic’ sociality and politics that displaces responsibility, mutual obligation, and reciprocity – the very forms that many social scientists argue trust takes – and, in the process, eviscerates trust as a contemporary social form. While acknowledging these critiques, our interest resides in being ethnographically attuned to the relations, infrastructures, and practices that such critiques are putatively built upon. The task at hand for this special issue, then, is to probe forms of (mis)trust within particular sets of digitally mediated relationships, putting ethnographic flesh on the bone, as it were, of such sweeping critiques. What we observe through our contributors’ work is that it is less a question of the erosion, dissipation, or suspension of trust as it is its mutation; *when (mis)trust arises as an issue of concern for actors as they problematize, narrate, scale, site, design, and code, it mutates through its materialization within different practices and protocols*. In the following, we provide a reading of the ethnographic encounters of our contributors as they intersect with specific computational forms.

## 4. Ethnographic encounters with digital (mis)trust

While critiques of surveillance capitalism take general aim at the deleterious effects of computational practices in contemporary societies, and particular aim at their impact on relations of trust, the burgeoning of ‘machinic’ modalities of social action seem almost endless. Instructive in this regard is the turn to algorithms and machine learning in the public sector. Here we see digitalization’s promises seeping into the beating heart of citizen-state relations. And for harried bureaucrats concerned with the provision of cheaper and more efficient state services, the promise of tools that claim to classify, categorize, and organize our worlds into knowable, and hence actionable, variables, is promise enough. We are increasingly witnessing various forms of algorithmic governance mechanisms entering into the frame within the justice system: sentencing and parole (Christin 2018), social services: child protection (Ratner and Elmholt 2023) and unemployment benefits (De Stefano 2020), urban planning: ghettos (Birk & Elmholdt 2020), predictive policing (Meijer et al 2021), education: national school tests (Høvsgaard Maguire 2019), and health: disease prevention and eradication (Hoeyer 2023). Accompanying such ‘algorithmic ideology’ (Mager 2012) is a somewhat overwrought desire to eliminate uncertainty from institutional life, achieved, in part, by grounding public decisions beyond the perceived fallibility of human subjectivity. But as Wendy Chun has pointed out (2013; 2015), the curious, and problematic, correlation between ever-increasing uncertainty and the turn to ‘programmability’ remains a difficult knot to cut. The effort to generate *more certainty* through a proceduralization of citizen-state relationships only amplifies the production of new forms of uncertainty. Here we see another specific instance of the role that computational forms and digital technologies play in the amplification and mutation of (mis)trust. As a perceived antitode to the uncertainties of contemporary existence, the processes of formalization and proceduralization that these forms embed come with their own latencies. And it is these new forms and latencies – as they bleed into questions of (mis)trust – that some of our contributors take up.

Singh’s contribution draws us into questions of uncertainty in public data infrastructures through an examination of Aadhaar, India’s biometrics-based national identification number system. Here Singh invites us into the politics of visibility of these numbers, exploring, for example, some of the tensions in their ambiguous status as numbers to be publicly disclosed while simultaneously remaining confidential. As a powerful digital intervention into the lives of citizens, the Aadhaar story outlines how computational approaches to questions of trust produce their own vulnerabilities and uncertainties in the public sphere. What we learn is that the doubleness of these computational technologies as both sites of citizen recognition and citizen surveillance generates a series of challenges, breakdowns, and repair mechanisms that produce (mis)trust in recursive, uneven, ways. As a social form, (mis)trust mutates at various scales and sites of intervention, at times, sustaining trusting relationships, whilst at others, heightening mistrust. These mutations are, furthermore, not unidirectional: their recursive form affords two-way traffic. Likewise, Gad’s contribution brings us into questions of citizens’ trust in state-democratic processes by homing in on the Danish debate over the digitalization of election processes. As he notes, the Danish public sector is consistently ranked as one of the most digitalized in the world, while simultaneously, the country also has some of the highest election participation rates. So, despite trust in traditional voting systems being extremely high, the political zeal for digitalization, Gad shows, led to a paradoxical effort to ‘*fix unbroken (voting) systems by digital means.’* By charting the positions of computer scientists and cryptographic experts in this debate, Gad highlights the mediating role of computational expertise in public discussions of trust, inviting us to consider some of the specific ways that computational forms of thinking move out of the academy and into issues residing at the heart of democracy and public debate.

Breslin’s contribution to the special issue presents an ethnography of computer science students in Singapore. Here, trust in more recognizable social forms and entities (people, institutions, disciplines) mutates into trust in numbers, math, and code. As students move through their studies, they encounter mistrust as an omnipresent concept, not just rhetorically and practically at sites of pedagogical training and assessment (lectures, exercises, presentations, exams) but as an aesthetic form in the structure of coding itself. By aesthetic, Breslin is referring to how particular modes of writing code are deemed (in)appropriate by others, and hence are deemed to have allayed or produced mistrust. Managing mistrust requires the development of an entire coding infrastructure of relations that valorizes becoming a ‘good programmer’ through the production of ‘trustworthy code.’ Although trustworthy here is not a cognitive assessment of the intentions and interests of other people (as one finds in classic understandings of the term), neither is it about the verifiable mathematical proofs one finds in, for example, cryptography (as we shall see in the next section). It is rather a question of cultivating a form of coding subjectivity through which students develop an aesthetic sense of what trust means and how it is encoded through disciplinary protocols. At the same time, Breslin points out that the broader social and material contexts through which such code is produced are rendered invisible to students, and therefore, in some sense, purify these future experts from valuable lessons about the socially embedded nature of coding.

But questions of digitally mediated (mis)trust not only arise through citizen-state encounters, or citizens’ enclosure within surveillance capitalism ecosystems. They reside very much within the heart of the ‘internet complex’ (Crary 2022). Digital infrastructures and their architectures of circulation (code, protocols, standards, platforms) not only set the stage for (mis)trust to arise within particular configurations, but more strongly, contribute to the conditions and structures for what constitutes (mis)trust in digital interactions. The very disciplines and practices connected to the secure communications traditions are emblematic in this regard. Cryptography, for example, assumes that digital architectures are fundamentally hostile communication environments, populated by malign actors ranging from nefarious individuals to state institutions. Approaching these architectures as such enacts them as spaces where the pre-conditions for trusting relationships (commonly thought of as forms of acquaintance or familiarity in western contexts) are undermined. Put differently, the idea that there is some shared implicit knowledge about the nature of actors and their relationships in digitalized environments is put into radical doubt. In the absence of such shared knowledge, cryptographers inscribe a technicalised version of these trust conditions within architectural environments, developing mathematical forms of authenticity (verifying actors’ identity), integrity (verifying that data has not been manipulated), and confidence (verifying their data remains invisible) that putatively produce trust through verification measures. Here mistrust is a method. It is not the absence of trust, but a mutation of it: trust is rendered calculable through its translation from a social problem to a technical form. By embedding these relationships in a framework of mathematical clarity (mathematically provable protocols) the problem of general mistrust in digital environments is rendered computational as *verifiable* trust (Ruh 2018), or what Werbach calls ‘trustless trust’ (2018, 28–29).

We see similar questions arise in the contributions by both Breslin and Gad where *mistrust as method* is made visible in computer science education and computational thinking in public policy. In the former, mistrust is omnipresent and becomes the basis upon which coding infrastructures, and hence trustworthy code, are developed. At the same time, trust is aestheticized as part of subjectivity formation. In the latter, computer scientists mobilize general mistrust as a method of highlighting the inadequacies of digitalized elections. In the Danish case, their intervention became a successful bulwark against the development of such systems. But as Gad interestingly points out, this does not hinder these computer scientists from envisioning a future where the perceived deficiencies of digitalized elections cannot be resolved, it ‘merely’ requires redesigning the entire elections process in the form of an algorithmic machine. In such a rendering, there is not a division between ‘trust in people’ and ‘trust in technology’ as oftentimes postulated in technology circles (see Singh’s contribution), but a reimagining of humans in the loop (election officials) as machinic nodes in an otherwise algorithmically oriented system. Here voting – a central democratic ritual – becomes computationally structured through rigorously engineered verification systems, the application of queuing theory to the design of polling stations, protocol design and analysis, and so on. Trust, in this future, is not eviscerated or suspended but has entirely mutated into a cybernetic expert vision of voting as democratic truth production.

Spencer’s contribution brings us into another tradition within secure communications communities: the more quotidian, but no less interesting, world of cyber security assurance. In this context, assurances are processes of certification for many digital products embedded within digital infrastructures (routers, switches, firewalls, operating systems, VPNs, and so on). What Spencer outlines is how various narratives trafficked between cyber security experts cast doubt on the trustworthiness, and hence value, of such formal certification schemes. All of these schemes, Spencer recounts, can be gamed, subverted, or manipulated to varying degrees and the stories that experts use to characterize them are what enact mistrust in this setting. Here mistrust is neither the pure absence of trust nor a disposition of persons; it takes mutable form through the agency of stories to act upon the capacity of other texts, decisions, and the future arc of new policy initiatives.

## 5. Mutations of digital (mis)trust

This special issue is driven by a curiosity about relations of (mis)trust under emerging conditions of digitalization. The encroaching digitalization of societies is, in many ways, an investiture in computational forms (practices, logics, technologies, and infrastructures) as a means of doing sociality. The computational mediation of interpersonal relations, social actions, public life, and essential infrastructures has far reaching implications, not least for who, what, and how we trust. In this sense, the rise of such forms amplifies, reconfigures, and, we argue, mutates, (mis)trust. The contributors to this special issue have highlighted not only an expansive set of empirical contexts where computational forms have become important mediation points for considering questions of (mis)trust in social life. They also suggest how these various forms reconfigure the conditions through which (mis)trust is constituted (what structures what counts as trust and mistrust) as well as the *mutability* of (mis)trust upon its entry into computational arrangements. Whether it is the role of computational thinking within public discourses on democratic elections (Gad), the use of computational technologies in attempts to establish bureaucratic order (Singh), the computational practices at play in the production of coding subjectivities (Breslin), or the computational artefacts that assure data circulations within digital infrastructures (Spencer), what this issue encourages us to reflect upon is the pervasive role of computational processes in social life and their importance as mediators of (mis)trust. Each article in the special issue makes its own contribution through the analysis of a set of ethnographic encounters. What these encounters highlight is that when people are confronted by rapidly changing (computational) landscapes, they tend to evoke trust and/or mistrust as an idiom to say that important matters are at stake. But the very articulation of these matters as ones of trust and mistrust impacts how they are navigated: these concepts organize ‘how we see’ those encounters (Harper 2014). Be it Singaporean coding students learning the tools of their craft, Indian citizens navigating a complex, ambiguous, and multi-valent data infrastructure, cyber-assurance experts trafficking in uncertain narratives about architectural protocols, or the Danish polity debating the role of cryptography in democratic processes. In essence, each ethnographic example highlights an encounter between a given set of actors (a polity, experts, students, citizens) and a particular set of computational forms (practices, logics, technologies, infrastructures). Within the space of these encounters (mis)trust is, in one way or another, problematized, conceptualized, debated, narrated, designed for, and so forth. And it is here that this introduction has set its analytical crosshairs, arguing for a more expansive understanding of the relations between trust and mistrust, less as antonyms, and more as co-constitutive, entangled, and mutable. In many of the aforementioned encounters, we notice that computational forms intervene in socio-political and cultural discussions, as that which can, in some sense, remedy what is perceived as problems of mistrust. Clearly, this is part of a technosolutionist approach to problem solving more generally, but the very analytical rendering of mistrust *as a problem* is part of what this special issue speaks to. It is our argument that conceptualizing the relation between trust and mistrust as antonyms does a form of work that goes beyond the boundaries of academic theorizing, entering into the spaces of encounter where actors frame troublesome social questions as *solveable*. It is within these spaces we see a rush by actors to resolve, solve, or in some sense ‘fix’the *problem of mistrust*. Simultaneusly, the analytical critique of this zeal is to suggest that trust is suspended, displaced, or eviscerated as a social form. What we want to signal is that despite the prevailing technosolutionist wisdom, and the social science critique of this impulse, the question of mistrust cannot be ‘solved’ (i.e. made to disappear) in socio-technical relations. But it can (and does) change shape and reappear in other forms. Whatever actors (engineers, programmers, professionals, etc.) do in their efforts to ‘fix’ mistrust, it mutates. Mutation, in this sense, signals a ceaselessness of change that continues on as actors develop new practices, devices, designs, and problems. New iterations come and go (techniques, apparatuses, platforms, concepts) but trust and mistrust continue as social forms, albeit differently across contexts. This is not a normative statement. It is not to suggest that we should not intervene in the many and varied ailments that accompany computational forms and digital technologies. Our sense is that understanding the mutable nature of trust and mistrust as social forms – (mis)trust – is a first step towards doing our interventions otherwise.

As to the form of such mutations, that is a highly contextual empiral question. From Breslin we see how the logic attributed to defensive coding – which assumes the fallibility of other coders – is deeply embedded in coding subjectivities. Here we learn how this mutates into mistrust in people and institutions, which subsequently *mutates* into trust in math and numbers. For Gad, we see how the attempt to overwrite trust in analog democratic proceses through a zeal for digitalized solutions mutates into public mistrust of digital elections, which again mutates into trust in cybernetic truth making for cryptography specialists. For Singh, we learn how mistrust in citizen intentions mutates into trust in data, which mutates once more into mistrust in personal numbers as debates flare up. And finally, for Spencer, we get some insight into how mistrust in certification schemes mutates into narrative devices that act on other texts and decision spaces to create future policy initiatives. Our collective rejoinder, therefore, is to suggest that trust and mistrust live in a conjoined relationship: (mis)trust rarely reaches an end point, it rarely ceases, but it does mutate.

## Notes

1 Harper comments that we need only think about the infelicitous term ‘cloud’ as a means of describing the internet’s architecture.

2 In the time-space between developing this special issue and its publication, AI has garnered a huge wave of attention, not least with the release of Chat-GPT and similar large language models. And while AI clearly raises many trust-related questions (e.g. Laux et al. 2024), it is somewhat beyond the scope of our framing to engage substantively with it.

3 Advanced Research Projects Agency Network.

4 It is important to say that this somewhat innocuous term, ‘promise to pay’, is in many ways an arch-metaphor for translating the theory of contract into online life.

5 Disintermediation technologies are those that eliminate the need for intermediaries in digital transactions. A commonly used example within cryptocurrency discourse is how to eliminate the role of banks within payment systems infrastructures.
